# Part II: Accuracy of Teledermatology in Skin Neoplasms

**DOI:** 10.3389/fmed.2020.598903

**Published:** 2020-11-23

**Authors:** Mara Giavina-Bianchi, Maria Fernanda Dias Azevedo, Raquel Machado Sousa, Eduardo Cordioli

**Affiliations:** Department of Telemedicine, Hospital Israelita Albert Einstein, São Paulo, Brazil

**Keywords:** teledermatology, telemedicine, accuracy: skin neoplasms, skin cancer, benign skin lesions, malign skin lesions, tele-health

## Abstract

Teledermatology has been proving to be of great help for delivering healthcare, especially now, during the SARS-CoV-2 pandemic. It is crucial to assess how accurate this method can be for evaluating different dermatoses. Such knowledge can contribute to the dermatologists' decision of whether to adhere to teledermatology or not. Our objective was to determine the accuracy of teledermatology in the 10 most frequent skin neoplasms in our population, comparing telediagnosis to histopathological report and in-person dermatologists' diagnosis. A retrospective cohort study was conducted in São Paulo, Brazil, where a store-and-forward teledermatology project was implemented under primary-care attention to triage surgical, more complex, or severe dermatoses. A total of 30,976 patients presenting 55,012 lesions took part in the project. Thirteen teledermatologists who participated in the project had three options to refer the patients: send them directly to biopsy, to the in-person dermatologist, or back to the general physician with the most probable diagnosis and management. In the groups referred to the in-person dermatologist and biopsy, we looked for the 10 most frequent International Statistical Classification of Diseases and Related Health Problems-10 (ICD-10) of skin neoplasms, which resulted in 289 histopathologic reports and 803 in-person dermatologists' diagnosis. We were able to compare the ICD-10 codes filled by teledermatologists, in-person dermatologists, and from histopathological reports. The proportion of complete, partial, and no agreement rates between the in-person dermatologist's, histopathologic report, and the teledermatologist's diagnosis was assessed. We also calculated Cohen's kappa, for complete and complete plus partial agreement. The mean complete agreement rate comparing telediagnosis to histopathological report was 54% (157/289; kappa = 0.087), being the highest for malign lesions; to in-person dermatologists was 61% (487/803; kappa = 0.213), highest for benign lesions. When accuracy of telediagnosis for either malign or benign lesions was evaluated, the agreement rate with histopathology was 70% (kappa = 0.529) and with in-person dermatologist, 81% (kappa = 0.582). This study supports that teledermatology for skin neoplasms has moderate accuracy. This result reassures that it can be a proper option for patient care, especially when the goal is to differentiate benign from malign lesions.

## Introduction

As we face this pandemic time around the world, telemedicine has been proving to be of great help for delivering healthcare. Any medical specialty that is based on image analysis, such as dermatology, is especially suitable for this method of care. Teledermatology has the potential to improve access to subspecialty expertise, reduce healthcare costs, and improve the overall quality of care. The three main teledermatology delivery platforms are: synchronous (RT: real-time teledermatology), asynchronous (SF-TD: store-and-forward teledermatology), and hybrid (both synchronous and asynchronous forms). Synchronous teledermatology uses live video conferencing between the patient and the dermatologist. Asynchronous teledermatology is a method whereby clinical or dermoscopy dermatologic images are obtained and sent to the responding dermatologist who can review them at a later time. Although it provides high-resolution dermatologic images and promotes an efficient practice, this modality is limited by the ability of the teledermatologist to obtain additional clinical history while evaluating the case (1). Teledermoscopy involves the use of dermoscopic images for remote consultation and decision-making in skin cancer screening. Its addition significantly improved the results of an internet-based skin cancer screening system, compared with clinical images alone (2).

The majority of studies in teledermatology have found rates of accuracy to be in the range of 75–80%, comparable to those with in-person care (1). Many of the articles were focused on skin neoplasms, especially skin cancer and pigmented lesions (3–6), or on general dermatology (7–11). A recent systematic review concluded that robust implementation studies of teledermatology are needed, with attention to reducing the risk of bias when assessing diagnostic accuracy (3). For this purpose, we performed research with the primary goal of determining the accuracy of teledermatology for skin neoplasms in a robust number of cases, assessing the agreement rate between the histopathological report, in-person dermatologists', and the teledermatologists' diagnoses. The secondary aim was to analyze the differential diagnosis of the lesions.

## Materials and Methods

This was a retrospective cohort study designed to assess concordance between diagnoses made by in-person dermatologists and teledermatologists, and by histopathological reports and teledermatologists, approved by the Ethics Committee of Hospital Israelita Albert Einstein (CAAE: 97126618.6.0000.0071). We analyzed the reports of 30,976 patients included in a teledermatology triage project conducted in the city of São Paulo, Brazil, from July 2017 to July 2018.

### Teledermatology Triage Project

**Figure 1:**
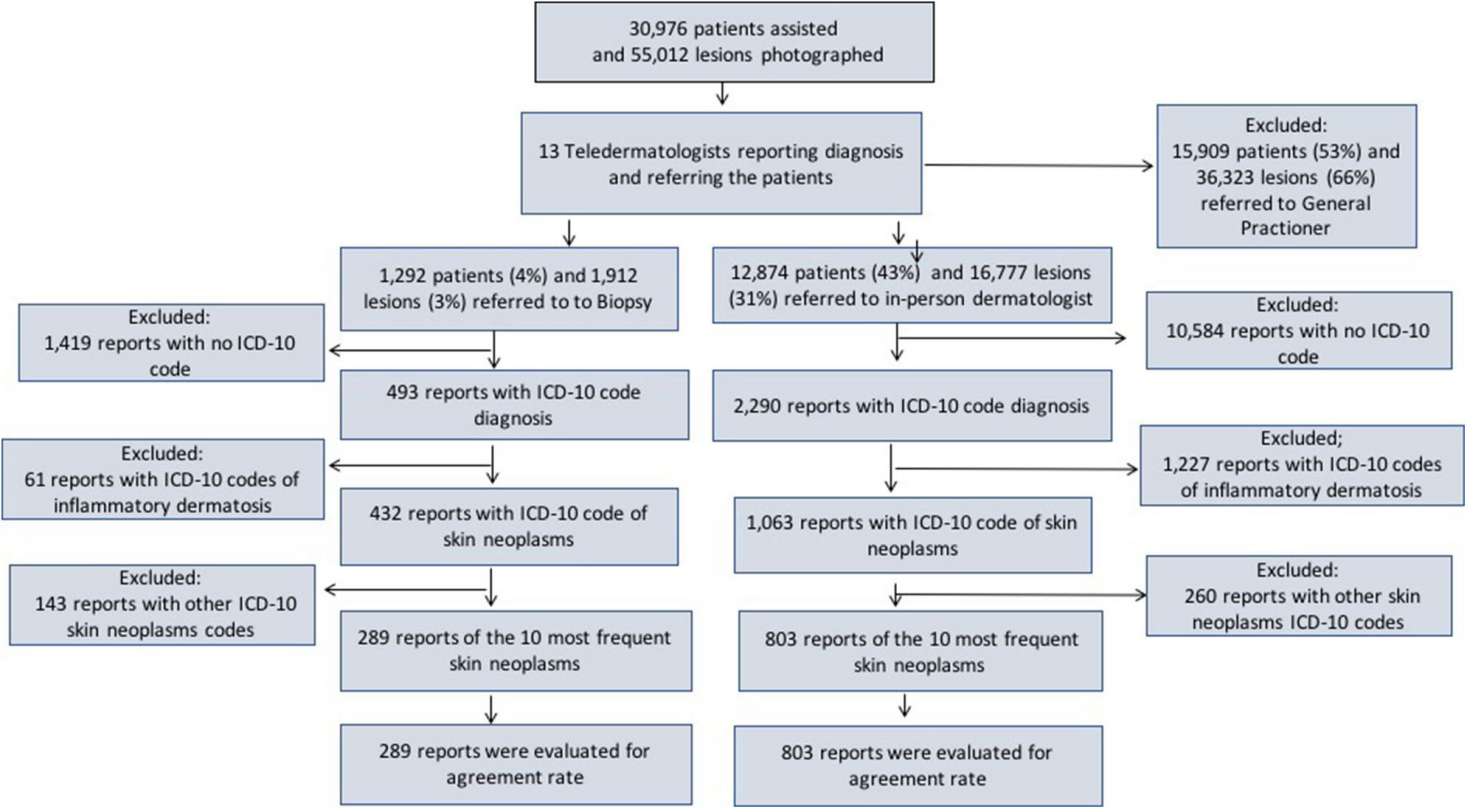
Frequency of patients included, photographed lesions and referrals made by the teledermatologists, along with the flow used to assess the diagnosis accuracy for the 10 most frequent skin neoplasms.

A Teledermatology project implemented from July 2017 to July 2018, in São Paulo. There were 57,832 individuals waiting for a consultation with a dermatologist in the public health system in July 2017. To reduce the waiting list and accelerate the flow of patients with the most severe, complex or surgical dermatoses to in-person dermatologists, the municipal health government requested Hospital Israelita Albert Einstein, a large private hospital in the city with expertise in Telemedicine, to develop a project using Telemedicine to reach these goals. Therefore, an online platform and a mobile app were designed to take photographs with phone cameras and directly upload them to the app and a short clinical history and data of the patient, which were meant to be used by for health technicians or nurses. The photographs and collected information were uploaded to a platform using a secure online process and were accessed only by the 13 Brazilian Board Certified dermatologists associated with this project, for whom the patients were randomly assigned. These 13 dermatologists will be called teledermatologists in this study. All the patients waiting for dermatologist consultations were called consecutively, via phone, by the municipal health care service and scheduled for an appointment in one of the three public city hospitals enabled to carry out the project, depending on the location of the individual's residence. Once there, a short history of the complaint (history of bleeding, pruritus, time from the onset, and location) and demographic data (sex and age) as well as three photographs in different angles and distances from each lesion were taken by a trained health technician: first one at medium distance (50 cm away), second one in close-up (15 cm away) and last one of a lateral view, aiming to evaluate the volume of the lesion. After accessing the patients' pictures and clinical history of the patients, the dermatologists first decided whether the photographs of the lesions were satisfactory for diagnostic purposes. If not, they categorized it under “bad photo,” and the patient was referred to a dermatologist for an in-person appointment. If the photo quality was good enough, they formulated the most probable diagnostic hypothesis. They chose one of three options for each lesion assessed: (1) a direct referral for a biopsy (after which the patient would return for an in-person dermatologist appointment), (2) a referral for an in-person dermatologist (IPD) visit, or (3) a referral to go back to the primary care physician (GP) with the most probable diagnosis, treatment and/or recommendation on how to proceed with the investigation and/or management of the lesion. A schematic for this process is shown in Figure 1. If the same patient had more than one lesion but different referrals, they were referred to the most specialized one and that would be his/her last referral in terms of statistical accounting. For instance, a biopsy would prevail over a dermatologist visit, which would in turn prevail over general physician referral. This teledermatology project followed the American Medical Association telemedicine policy, adapted for the needs of the Brazilian public health care system (12).

### Study Design

We selected the groups referred to IPD (12,874 patients/16,777 lesions) and biopsy (1,292 patients/1,912 lesions). Then, we assessed the reports that had the International Statistical Classification of Diseases and Related Health Problems 10th version (ICD-10 code) (13) diagnoses filled by IPD (2,290) and histopathological reports (HP: 493). Next, we separated the reports filled with ICD-10 codes of skin neoplasms sent to IPD (1,063) and from histopathological reports (432). Afterward, we looked for the 10 most frequent dermatoses in each group, 803 in IPD and 289 to include in our study (803 for IPD and 289 in HP Figure 1).

We classified the rate of agreement as follows: (1) complete agreement when the ICD-10 code used in both reports were the same, (2) partial agreement when the ICD-10 code used in both reports were different, but in the same group of disease (malign or benign neoplasms), posing as a probable differential diagnosis, and (3) no agreement when both reports did not fill the previous two conditions. Basal cell carcinoma (BCC), squamous cell carcinoma (SCC), melanoma, and actinic keratosis (AK) were considered the malign group. Nevus, seborrheic keratosis (SK), acrochrodon/other benign neoplasms, cyst, lipoma, and wart were the benign group. If the cases did not belong to one of those 10 classes, it was classified as “other.” As the gold standard diagnosis is the histopathological diagnosis, the agreement was stated in this research as accurate.

### Statistical Analysis

Rates of concordance were expressed using percentages and Cohen's kappa coefficient, which was used to compare between groups of inter-rater observers (Graph Pad Prism 6.0). The guidelines used to characterize kappa values were created by Landis and Koch (14), as follows: kappa <0: no agreement, 0.00–0.20: slight agreement, 0.21–0.40: fair agreement, 0.41–0.60: moderate agreement, 0.61–0.8: substantial agreement, and 0.81–1.00: almost perfect agreement.

## Results

Table 1 shows the 10 most frequent skin neoplasms diagnosed by teledermatology according to the frequency of lesions, sex, age, and referral distribution. The total amount represents 31% (17,233/55,012) of the lesions diagnosed in the entire teledermatology project. The female and male proportion was 72 and 28%, respectively, although the female population accounts for 52.6% in the city of São Paulo (2010) (15). Lesions diagnosed as benign were 90% (15,496/17,233) and as malign, 10% (1,727/17,233). Among benign neoplasms, 63% (9,849/15,496) were sent to GP for follow-up, 34% (5,242/15,496) were sent to IPD and only 3% (405/15,496) were sent to biopsy. For malign lesions, 3% (4/1,727) was referred to GP, 51% (880/1,727) to IPD and 46% (803/1,727) to biopsy.

**Table 1:** Patient's demographics and referrals for the 10 most frequent skin neoplasms diagnosed in the Teledermatology Project in São Paulo from July 2017–2018.

	**Sex**		**Age (years)**			**Referral**			
**Skin neoplasm**	**M**	**F**	**0–19**	**20–59**	**60+**	**Biopsy**	**IPD**	**GP**	**Total**
BCC	144	274	0	154	264	341	73	4	418
SCC	120	188	5	101	202	267	39	2	308
Melanoma	45	118	7	68	88	117	46	0	163
AK	280	558	2	190	646	78	722	38	838
Nevus	917	3,434	399	2,985	967	166	1,491	2,694	4,351
SK	784	2,251	4	1,124	1,907	75	507	2,453	3,035
Acro/other	889	2,872	275	2,483	1,003	76	539	3,146	3,761
Cyst	738	1,176	119	1,172	623	41	1,059	814	1,914
Lipoma	394	510	26	590	288	3	362	539	904
Wart	592	939	489	722	320	44	1,284	203	1,531
Total	4,903	12,320	1,326	9,589	6,308	1,208	6,122	9,893	17,233

**Figure 2:**
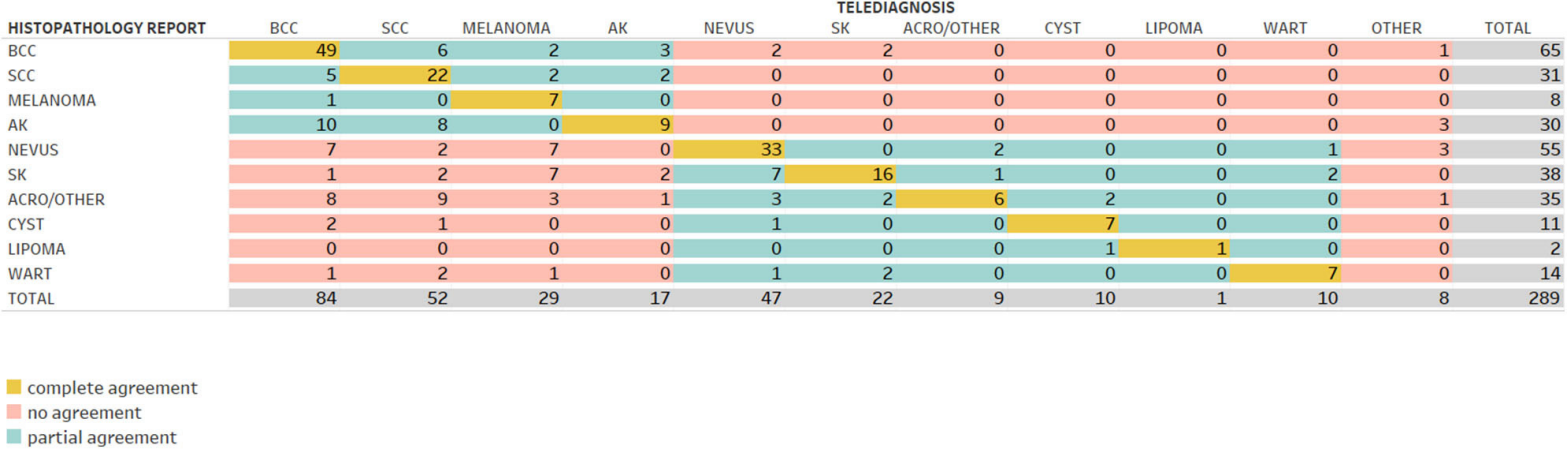
Confusion matrix comparing telediagnosis to histopathology report.

**Figure 3:**
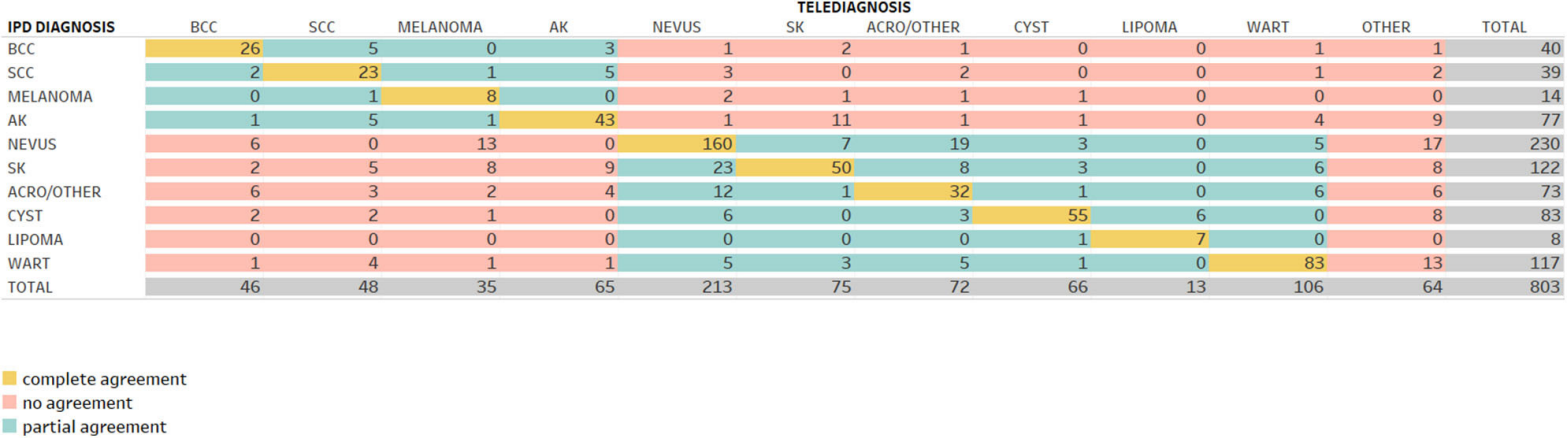
Confusion matrix comparing telediagnosis to in-person dermatologists' diagnosis.

Figures 2, 3 demonstrate the comparison between teledermatologists' diagnosis to the histopathological reports and to in-person dermatologists, respectively.

For instance, in Figure 2, among the 65 histopathological reports of BCC in this study, the teledermatologists had diagnosed 49 correctly (complete agreement in yellow), 6 as SCC, 2 as melanoma, 3 as AK (all considered partial agreement, in green), 2 as nevus, 2 as SK, and 1 as other (all considered no agreement, in red).

Following the same path, in Figure 3, among 40 telediagnosis of BCC, 26 were in agreement with IPD (complete agreement, in yellow), 5 were SCC, 3 were AK (partial agreement, in green) and 1 nevus, 2 SK, 1 acro/other, 1 wart, and 1 other (no agreement, in red).

Table 2 reveals the percentage of accuracy for complete, partial, and no agreement and Cohen's kappa calculations for complete and complete plus partial agreement, comparing the telediagnosis to histopathological reports (upper part) and in-person dermatologists' diagnosis (bottom part). In the upper part, the mean frequency of complete agreement was 54% for all 10 dermatoses tested (157/289) and its kappa coefficient was 0.087), which is considered a slight agreement. Three malign skin tumors had the highest rates of accuracy: melanoma (7/8), BCC (49/65), and SCC (22/31), respectively with 88, 75, and 71% and kappas = 0.060, 0.326, and 0,117. Among benign neoplasms, cyst, and nevus were the most accurate diagnoses, respectively with 64 and 60% and kappas = 0.335 and 0.298 (fair agreement). Warts, SK and lipomas showed an intermediate rate, around 50%, and acrochordon and other benign neoplasms had the lowest rate (17%; kappa = −0.078). AK had only 30% of agreement, being the least accurate in the malign group (kappa = −0.146). Partial agreement was verified in 22% of all cases (64/289), ranging from 5% in nevus up to 60% in AK. No agreement was found in 24% (68/289); CBC, SCC, and lipoma had zero no agreements while nevus, SK, warts, and cysts had around 30% of no agreement.

**Table 2:** Agreement between teledermatologists' diagnosis and histopathological reports (upper part) and in-person dermatologists (bottom part).

	**Teledermatologists' diagnosis**				
**Histopathology**	**Complete agreement**	**Cohen's Kappa**	**Partial agreement**	**Cohen's Kappa complete**	**No agreement**
**report**	**(%)**	**complete agreement**	**(%)**	**+ partial agreement**	**(%)**
BCC	75	0.326	17	0.680	8
SCC	71	0.117	29	0.627	0
Melanoma	88	0.060	12	0.209	0
AK	30	−0.146	60	0.724	10
Nevus	60	0.298	5	0.596	35
SK	42	0.129	26	0.513	32
Acro/other benign	17	−0.078	20	0.195	63
Cyst	64	0.335	9	0.717	27
Lipoma	50	0.400	50	1.00	0
Wart	50	0.189	21	0.676	29
Total	54	0.087	16	0.529	30
**In-person dermatologists' diagnosis**					
BCC	65	0.213	20	0.472	15
SCC	59	0.067	20.5	0.496	20.5
Melanoma	57	−0.138	7	−0.050	36
AK	56	0.217	9	0.427	35
Nevus	70	0.446	15	0.807	15
SK	41	0.068	34	0.527	25
Acro/other benign	44	−0.117	27	0.642	29
Cyst	66	0.483	22	0.799	12
Lipoma	87.5	0.369	12.5	1.00	0
Wart	71	0.490	12	0.768	17
Total	61	0.213	20	0.582	19

Comparing teledermatologists' to in-person dermatologists' diagnosis accuracy, we found a mean frequency of complete agreement in 61% of all 10 skin neoplasms (487/803) and its kappa coefficient was 0.213, which is considered a fair agreement. Benign skin tumors had the highest rates of accuracy: lipoma (7/8), warts (83/116), and nevus (160/230), with 87.5, 72, and 70% and kappas = 0.369, 0.490, and 0.446, respectively. Among malign neoplasms, all four of them were between 56% (AK) and 65% (CBC). The lowest rate was achieved by SK (41%; kappa = 0.068) and acro/other benign (44%; kappa = −0.117). Partial agreement was verified in 20% of all cases (159/803), ranging from 7% in melanoma up to 34% in SK. No agreement was found in 19% (156/803); lipoma had zero no agreements while melanoma, AK, and acro/other benign had around 36, 35, and 29%, respectively.

## Discussion

Analyzing Table 1, we can see that most of the benign cases of skin neoplasms in this cohort were not sent to biopsy and in-person dermatologists; they were sent back to the GP to follow-up and, therefore, could not be compared with the teledermatologists' diagnosis. This fact is very relevant when discussing accuracy in this study. As the teledermatology triage project prioritized the severe, more complex, or surgical cases for biopsy and in-person dermatologists, trying to manage the mild cases under the primary-care attention along with the GP, skin neoplasms diagnosed by histopathological analysis or in-person dermatologists have a bias of being the most challenging/difficult cases. Typical or “regular” skin neoplasms were most probably diagnosed and referred back to the GP. In this manner, our results in the rate of agreement would be probably much higher if more typical cases were analyzed.

In terms of health delivery, choosing the proper management of the skin neoplasms is even more important than elaborating the right diagnosis itself. In this way, what is more important than complete agreement (ICD-10 code) is to analyze rates of complete plus partial agreement (benign vs. malign). If the diagnosis fits within the benign neoplasms, an expectant conduct is acceptable, while within the malign neoplasms, a biopsy, or surgery ought to be expected.

Analyzing teledermatogists' diagnosis and histopathological reports' agreement, we showed a mean agreement rate of 70% (54% total + 16% partial agreement rates) and kappa = 0.529 (moderate). Considering studies that compared TD diagnosis and histopathological reports, Silveira et al. (16) included 364 suspected cases of skin malignancy by TD to be confirmed by the biopsy. The majority of the lesions were BCCs with 286 cases, followed by 59 SCCs and 5 melanomas. Two oncologists showed an overall accuracy of 85.3% for the first and 87.3% for the other in categorizing benign x malign lesions. Our results for malign and benign lesions showed 94% (126/134) and 61% (95/155) of agreement, respectively. In another study, 144 pigmented lesions diagnosed via TD were examined histologically, and 63 (43%) showed complete agreement between the clinical and histological diagnoses (17). Comparatively, our study showed 60 and 88% complete agreement for nevus and melanoma respectively. In another article, 201 biopsies were requested to rule out malignancy via TD, which was confirmed in 45.3% (18). A survey among the teledermatologists participating in the project, after its conclusion, showed that the use of dermoscopy would significantly improve the decision to refer nevus and SK to in-person dermatologists, GP or biopsy and, most probably, the agreement rate too. They felt that many cases referred to biopsy or IPD could have been referred to GP if dermoscopic images were available (19). Melanoma had a fair agreement rate. We believe that happened because, when in doubt, one would rather excise it and send it to histopathological than wait to see how it develops. In this project, with no access to dermoscopic images, there were 112 lesions suspected of melanoma and 8 melanomas confirmed (14 benign lesions:1 melanoma). Other studies show that the number of nevus needed to diagnose one melanoma can vary from 4.5 to 22 (20). Acrochordon/other benign neoplasms had slight agreement rate kappa, probably because of the reasons explained before. Only “weird looking” acrochordons would be sent to biopsy, confirming that they were, in fact, not acrochordons.

The results of our study showed a slightly better agreement rate between diagnoses made by teledermatologists and in-person dermatologists in general. If we summed up complete (61%) and partial (20%) agreement rates, we have reached 81% (kappa = 0.582-moderate), but there was an inversion in the accuracy for benign and malign lesions. The agreement rate for benign lesions was 82% and for malign, 73%. Nevus and acrochordons/other benign neoplasms in this referral were probably more typical than the ones sent to biopsy, and that could explain why the agreement rate for benign skin neoplasms had an improvement compared to the previous one. On the other hand, BCC, SCC, and AK showed lower agreement rate to IPD than histopathological reports. The worst agreement rate was for melanoma. Cohen's kappa coefficient showed no agreement (−0.050). This may reflect the same reasons stated before, the absence of dermoscopy images and also the teledermatologists' fear of letting a melanoma diagnosis go undetected. When compared to the previous studies, Kroemer et al. (21) included 104 pigmented lesions and found a kappa = 0.84. Moreno-Ramirez et al. (22) showed a kappa = 0.81 in 890 cases of skin cancer triage.

According to the latest Cochrane review (2018) about teledermatology in skin cancers, using a more widely defined threshold to identify “possibly” malignant cases or lesions that should be considered for excision is likely to appropriately triage those lesions requeiring face-to-face assessment by a specialist. Despite the increasing use of teledermatology on an international level, the evidence base to support its ability to accurately diagnose lesions and to triage lesions from primary to secondary care is lacking and further prospective and pragmatic evaluation is needed (5).

Although this was a retrospective study and much data was missing, we believe this was one of the studies with the largest number of skin neoplasms included in the literature, comparing teledermatologists' diagnosis both to IPD and histopathological reports. Pathologists were responsible for histopathological reports. The study was performed in two centers and different dermatologists performed the tele and in-person examinations. This is a limitation because there is interoperator variability and the technical skills in the groups may be different. Moreover, teledermatologists could diagnose the lesions differently if they were face-to-face. The profile of 12 out of the 13 teledermatologists involved in the project was shown in a previously study (19). Ten had either subspecialty in dermoscopy or skin cancer, which we thought was very suitable for this study. Six had more than 10 years as Board Certified Dermatologists. Five of them integrated the Skin Oncology outpatient unity at University of São Paulo Dermatology Department. Unfortunately, we do not have the profile of the IPD. The risk of false negative cases, which we could not assess, exists and it is another limitation of this research. Nonetheless, during the project, there was always a physician, either a GP or an IPD, responsible for monitoring the patients.

Our study, performed in a large number of patients presenting the 10 most common skin neoplasms, showed moderate accuracy between teledermatology and histopathological reports, and moderate agreement rate between teledermatologists and in-person dermatologists' diagnosis. Accuracy may differ among skin neoplasms. This reassures that store-and-forward teledermatology can be an option for triage skin neoplasms in primary-care attention.

## Data Availability Statement

The raw data supporting the conclusions of this article will be made available by the authors, without undue reservation.

## Ethics Statement

The studies involving human participants were reviewed and approved by Ethics Committee of Hospital Israelita Albert Einstein (CAAE: 97126618.6.0000.0071). Written informed consent from the participants' legal guardian/next of kin was not required to participate in this study in accordance with the national legislation and the institutional requirements.

## Author Contributions

MG-B and EC: study design. MG-B, RS, MA, and EC: data collection and review of the article. MG-B and MA: data analysis. MG-B and RS: writing. All authors contributed to the article and approved the submitted version.

## Conflict of Interest

The authors declare that the research was conducted in the absence of any commercial or financial relationships that could be construed as a potential conflict of interest.

## Abbreviations

TD: teledermatology
RT: real time
SF-TD: store and forward
IPD: in-person dermatologist
GP: general physician
ICD-10 code: international code of diseases 10
BCC: basal cell carcinoma
SCC: squamous cell carcinoma
AK: actinic keratosis
SK: seborrheic keratosis
Acro/other benign: acrochordon/other benign neoplasms.
